# An implementation strategy postmortem method developed in the VA rural Transitions Nurse Program to inform spread and scale-up

**DOI:** 10.1371/journal.pone.0298552

**Published:** 2024-03-08

**Authors:** Heather Gilmartin, Christine Jones, Mary Nunnery, Chelsea Leonard, Brigid Connelly, Ashlea Wills, Lynette Kelley, Borsika Rabin, Robert E. Burke

**Affiliations:** 1 Denver/Seattle Center of Innovation for Veteran-Centered and Value Driven Care, VA Eastern Colorado Healthcare System, Aurora, Colorado, United States of America; 2 Department of Health Systems, Management and Policy, Colorado School of Public Health, University of Colorado, Anschutz Medical Campus, Aurora, Colorado, United States of America; 3 Division of Geriatric Medicine and Division of Hospital Medicine, Department of Medicine, University of Colorado, Anschutz Medical Campus, Aurora, Colorado, United States of America; 4 Herbert Wertheim School of Public Health and Human Longevity Science, University of California San Diego, San Diego, California, United States of America; 5 Altman Clinical and Translational Research Institute, Dissemination and Implementation Science Center, University of California San Diego, San Diego, California, United States of America; 6 Center for Health Equity Research and Promotion, Corporal Crescenz VA Medical Center, Philadelphia, Pennsylvania, United States of America; 7 Hospital Medicine Section – Division of General Internal Medicine, University of Pennsylvania Perelman School of Medicine, Philadelphia, Pennsylvania, United States of America; 8 Leonard Davis Institute of Health Economics, University of Pennsylvania, Philadelphia, Pennsylvania, United States of America; Lorestan University of Medical Sciences, ISLAMIC REPUBLIC OF IRAN

## Abstract

**Background:**

High-quality implementation evaluations report on intervention fidelity and adaptations made, but a practical process for evaluating implementation strategies is needed. A retrospective method for evaluating implementation strategies is also required as prospective methods can be resource intensive. This study aimed to establish an implementation strategy postmortem method to identify the implementation strategies used, when, and their perceived importance. We used the rural Transitions Nurse Program (TNP) as a case study, a national care coordination intervention implemented at 11 hospitals over three years.

**Methods:**

The postmortem used a retrospective, mixed method, phased approach. Implementation team and front-line staff characterized the implementation strategies used, their timing, frequency, ease of use, and their importance to implementation success. The Expert Recommendations for Implementing Change (ERIC) compilation, the Quality Enhancement Research Initiative phases, and Proctor and colleagues’ guidance were used to operationalize the strategies. Survey data were analyzed descriptively, and qualitative data were analyzed using matrix content analysis.

**Results:**

The postmortem method identified 45 of 73 ERIC strategies introduced, including 41 during pre-implementation, 37 during implementation, and 27 during sustainment. External facilitation, centralized technical assistance, and clinical supervision were ranked as the most important and frequently used strategies. Implementation strategies were more intensively applied in the beginning of the study and tapered over time.

**Conclusions:**

The postmortem method identified that more strategies were used in TNP than planned and identified the most important strategies from the perspective of the implementation team and front-line staff. The findings can inform other implementation studies as well as dissemination of the TNP intervention.

## Introduction

High-quality implementation evaluations commonly report the fidelity of implementation of an evidence-based intervention, and how the intervention was adapted. However, it is far less common to systematically evaluate the extent to which planned implementation strategies were delivered as intended, and how they were adapted over the life-course of a study [1]. There is good reason to suspect that implementation strategies, just like evidence-based interventions, undergo adaptation, to maximize the “fit” in a particular context [2]. Further, different implementation strategies may be used or emphasized during different phases of a study rather than assuming that all strategies are used in the same way from pre-implementation through sustainment phases [3]. Without systematic assessment and documentation of what strategies were used, when, and how, it is difficult to understand why a particular intervention or implementation outcome was achieved and how this could be replicated [1].

Implementation scientists commonly identify specific or a bundle of implementation strategies *a priori* to support intervention implementation. Ideally, tracking implementation strategy adaptations–like intervention adaptations–would occur over the course of the study. One option is to use the Framework for Reporting Adaptations and Modifications to Evidence-based Implementation Strategies (FRAME-IS) method [4]. FRAME-IS builds on the FRAME method [2] for tracking intervention adaptation. FRAME-IS includes modules for team members and local implementers to document which implementation strategy is modified, the nature, primary goal, rationale, and timing for the modification, participants in the decision-making process, and the spread of the modification. FRAME-IS is designed to capture modifications to strategies identified *a priori*, but not those added during implementation of the study. Another option involves longitudinal tracking of implementation strategies using a timeline follow-back procedure during the course of a study [5]. The Longitudinal Implementation Strategies Tracking System (LISTS) tracks the use of implementation strategies identified *a priori* and during a study, including fidelity concerns and adaptations [5].

The FRAME-IS and LISTS methods are a significant contribution to the field that hold potential to promote rigor and reproducibility in implementation research. However, the problem is these methods are resource-intensive, do not capture the perspectives of the staff outside the implementation team, and do not capture potentially valuable information regarding which implementation strategies were perceived as most useful. In other words, while the FRAME-IS and LISTS methods capture how and why the implementation team adapted implementation strategies, they provide limited insight into the extent to which these changes were perceived by staff receiving the implementation strategy. In addition, the FRAME-IS and LISTS methods provide little guidance regarding how to evaluate implementation strategy fidelity and perceived importance once an intervention has moved to the sustainment phase.

Given this, our team developed an implementation strategy postmortem method, using a 3 year, 11-site implementation program as a case study [6, 7]. Our explicit goals were: 1) to identify and specify which implementation strategies were planned *a priori* and compare them to which strategies were used and when from the perspective of the implementing team and front-line staff (i.e., fidelity); 2) describe the importance, frequency and ease of use of the implementation strategies from these perspectives, and 3) understand the barriers and contributors to successful implementation, and the perceived targets of each strategy from the perspectives of the implementation team and front-line staff. We explicitly did not evaluate the “dose” of implementation strategies delivered, which is an important but distinct concept related to “how much” of a particular implementation strategy was received. Rather, we argue an initial evaluation should focus on which implementation strategies were used, when, by whom and the perceived importance. Once these are evaluated, then it may be appropriate to analyze the “dose” of those strategies used to aid in interpreting study outcomes.

## Materials and methods

We used an iterative mixed methods [8] approach to describe and thematically analyze the multiple phases of data collected. We use the Veterans Health Administration (VA) rural Transitions Nurse Program (TNP) as a case study.

### The rural Transitions Nurse Program intervention

The transition from VA hospitals to home is a high-risk period for rural Veterans. This group is particularly vulnerable to gaps in communication and fragmented care coordination, resulting in inadequate access to follow-up care and high rates of hospital utilization [9, 10]. To address these gaps, TNP, a nurse-led pre-and post-discharge care coordination intervention, was implemented at 11 urban VA hospitals that treated large numbers of rural Veterans [6]. This national hybrid-effectiveness study enrolled 3,001 Veterans between April 2017 to September 2019. Veterans enrolled in TNP were more likely to see their primary care provider within 14-days of discharge than 6,002 matched controls (Odds Ratio 2.24, 95% CI 2.05–2.45). TNP enrollment was not associated with reduced 30-day emergency department visits or readmissions but was associated with reduced 30-day mortality (Hazard Ratio 0.33, 95% CI 0.21–0.53) [7].

The TNP study design, implementation and evaluation methods have been previously reported [6, 11–16]. Briefly, TNP was implemented with one nurse and physician champion per site. The transitions nurse was hired and trained to identify high-risk Veterans and coordinate care for Veterans across care settings [7]. During pre-implementation, the TNP implementation team conducted site assessments and process mapping of transitions of care activities to learn local context and understand the workflow at each site [11–14]. Evaluation of TNP was guided by the Reach, Effectiveness, Adoption, Implementation, Maintenance (RE-AIM) Framework [17].

### Postmortem implementation strategy method

#### Specification

**Fig 1:**
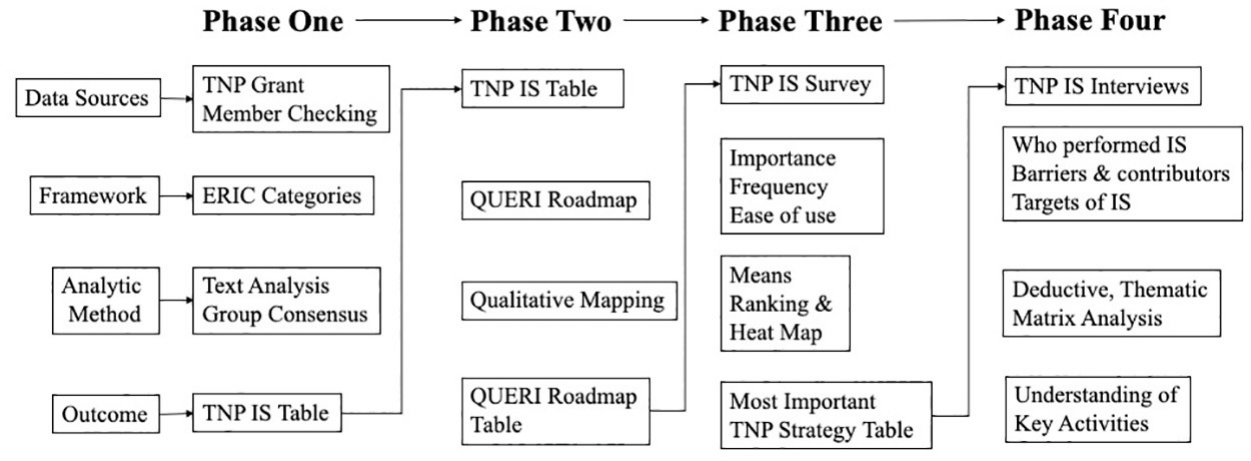
Postmortem method flow chart.

In 2019, the final year of the TNP study, we sought to enhance our understanding of the implementation strategies used, with guidance from the Expert Recommendations for Implementing Change (ERIC) compilation [18] and the Quality Enhancement Research Initiative (QUERI) Roadmap phases (pre-implementation, implementation, sustainability) [3]. Of note, the FRAME-IS [4] and LISTS [5] methods were not published at the time TNP was being implemented. A multi-phase approach was used that started with text analysis of the TNP grant application to identify the implementation strategies proposed *a priori* (Fig 1). All eleven members of the TNP implementation team were invited and engaged in a single member checking meeting to review the *a priori* implementation strategies and matched them to one of the 73 ERIC implementation strategies [18]. A follow-up meeting determined if other ERIC strategies were introduced during TNP. The purpose of this phase was to prompt reflection on the strategies originally outlined, identify if any were added as the program was implemented and sustained, and to label and define each strategy (S1 File).

In phase two, the list of TNP implementation strategies were qualitatively mapped to the QUERI implementation roadmap by the TNP clinical leads and principal investigator to identify when each strategy was used (Fig 1) [3]. The TNP pre-implementation phase was defined as the work conducted from the announcement of grant funding to sites hiring their first transitions nurse. The implementation phase was defined as the launch of the transitions nurse, site champion and leadership engagement efforts to 18 months post-implementation. The sustainment phase was defined as the final 18 months of grant funding, when no new sites implemented the intervention. The purpose of phase two was to understand the application and temporality of strategies over the life-course of the 3-year study [3, 19].

#### Ranking

In phase three, six TNP transitions nurses and site champions located at six VA medical centers were invited to participate in a survey to indicate the importance, frequency, and ease of use of the TNP implementation strategies identified in phase one. The six sites were purposely selected for they were currently implementing TNP at their sites. The purpose of phase three was to operationalize each strategy and gather perspectives of those who received the implementation strategy. The survey was developed per the specification categories outlined by Proctor et al. [19] and adapted for this project (S2 File). Each implementation strategy was paired with a TNP relevant definition (i.e., centralized technical assistance = In TNP, we developed and used the Denver-based team as a core group to deliver technical assistance focused on implementation issues). Importance was assessed by the question: “How important were the following implementation strategies in the implementation of TNP?” on an ascending five-point Likert scale (not important to very important). Frequency was assessed using the question: “How frequently were the following strategies used to implement TNP?” on an ascending five-point Likert scale (never to 3+times/week). Ease of use was assessed through the following question: “How easy were the following strategies to develop and use to support TNP implementation? (Please consider the time, effort, people, and materials required),” rated on a five-point ascending Likert scale (not easy to very easy). Item means were calculated for each strategy using Excel v16.59. The TNP implementation strategies were ranked by importance, frequency, and ease of use. The magnitude of difference within and between strategies were presented using a heat map data visualization technique designed in Excel, assigning a 5-color gradient to each Likert scale response [20].

In phase four, a convenience sample of eight members of the TNP implementation and clinical site teams participated in phone interviews to understand who delivered the strategies (i.e., the actor), barriers and contributors to successful implementation, and the targets of each strategy (i.e., the action target) [19]. Participants were asked about the top three most important and frequently used implementation strategies from phase three. Additional questions included the frequency of use and key activities that supported each strategy (S3 File). The interviews were recorded, transcribed verbatim, and analyzed in Excel using deductive, thematic matrix analysis [21]. TNP implementation team members not involved in the interviews double coded three transcripts to ensure coding consistency. When coding discrepancies arose, they were discussed and resolved. In accordance with VA institutional review board this study is a designated program evaluation. The postmortem results are presented in accordance with the Standards for Quality Improvement Reporting and Excellence Guidelines [22].

## Results

In phase one, four implementation strategies were listed *a priori* to support TNP implementation: pre-implementation site assessment, intervention training, audit and feedback, and facilitation [15, 16]. These matched the following ERIC-defined strategies: conduct local needs assessment, conduct ongoing training, audit, and provide feedback, and facilitation. The member checking exercise identified an additional 41 implementation strategies, resulting in a total of 45 ERIC strategies used in TNP. Phase two analyses indicated most TNP strategies (n = 22; 49%) were applied across the three QUERI Roadmap phases, while some were applied only during pre-implementation and implementation phases (n = 10; 22%) or only during the pre-implementation phase (n = 8; 18%). Implementation and sustainment phases (N = 4; 9%) or sustainment phase alone (N = 1; 2%) were less common (Table 1).

**Table 1:** TNP implementation strategies and QUERI Roadmap phases.

TNP Implementation Strategy	QUERI Roadmap Phase		
	Pre-implementation	Implementation	Sustainment
**Assess for readiness and identify barriers and facilitators.**	**x**		
**Audit and provide feedback.**		**x**	**x**
**Build a coalition.**	**x**	**x**	
**Capture and share local knowledge.**	**x**	**x**	**x**
**Centralize technical assistance**	**x**	**x**	**x**
**Change record systems.**	**x**		
**Conduct educational meetings.**	**x**	**x**	**x**
**Conduct educational outreach visits**	**x**	**x**	**x**
**Conduct local consensus discussions.**	**x**	**x**	**x**
**Conduct local needs assessment**	**x**		
**Conduct ongoing training.**	**x**	**x**	**x**
**Create a learning collaborative.**	**x**	**x**	**x**
**Develop academic partnerships.**	**x**	**x**	**x**
**Develop and implement tools for quality monitoring.**	**x**	**x**	**x**
**Develop and organize quality monitoring systems.**	**x**	**x**	**x**
**Develop educational materials.**	**x**	**x**	
**Distribute educational materials.**	**x**	**x**	
**Develop resource sharing agreements**	**x**		
**Facilitate relay of clinical data to providers.**	**x**	**x**	**x**
**Facilitation**	**x**	**x**	**x**
**Fund and contract for the clinical innovation.**	**x**	**x**	**x**
**Identify and prepare champions**	**x**		
**Inform local opinion leaders.**	**x**	**x**	**x**
**Intervene with patients/consumers to enhance uptake and adherence**		**x**	**x**
**Involve executive boards.**			**x**
**Make training dynamic.**	**x**	**x**	**x**
**Model and simulate change.**	**x**		
**Obtain and use patients/consumers and family feedback.**	**x**	**x**	
**Organize clinician implementation team meetings.**	**x**	**x**	**x**
**Prepare patients/consumers to be active participants.**		**x**	**x**
**Promote adaptability.**	**x**	**x**	**x**
**Promote network weaving.**	**x**	**x**	**x**
**Provide clinical supervision.**	**x**	**x**	**x**
**Provide local technical assistance.**	**x**		
**Provide ongoing consultation.**	**x**	**x**	
**Purposefully reexamine the implementation**		**x**	**x**
**Recruit, designate, and train for leadership.**	**x**	**x**	**x**
**Remind clinicians.**	**x**	**x**	
**Revise professional roles.**	**x**	**x**	**x**
**Shadow other experts.**	**x**	**x**	
**Stage implementation scale up**	**x**		
**Tailor strategies.**	**x**	**x**	
**Use an implementation advisor.**	**x**	**x**	**x**
**Use data experts.**	**x**	**x**	**x**
**Use data warehousing techniques.**	**x**	**x**	

In phase three, surveys were received from five TNP transitions nurses and one site champion from 6 VA facilities (100% response rate). Facilitation was rated very important (mean: 5) to implementation. An additional 30 strategies were rated as important (mean: 4.0 to 4.9) (e.g., centralize technical assistance, use data experts) and 14 as neutral (3.0–3.9) (e.g., tailor strategies, shadow other experts). No strategies were identified as less or not important. Facilitation was the most frequently used strategy (mean 4.7; 1-3x/week), followed by 12 strategies used 1-2x month (e.g., centralize technical assistance, create a learning collaborative), 31 strategies used 1-2x a year (e.g., remind clinicians, distribute educational material), and one strategy that was never used (i.e., conduct local consensus discussions). None of the strategies were rated as very easy or easy to use or develop.

Fifteen strategies earned neutral responses (e.g., ongoing consultation, develop resource sharing agreements), 29 were less easy (e.g., promote adaptability, build a coalition), and one was deemed not easy to develop or use (i.e., obtain and use patients/consumers and family feedback). Facilitation was identified as the most important and frequently used TNP strategy, followed by centralized technical assistance and provide clinical supervision. All three were acknowledged as less easy to develop and use then other strategies including use of data experts and educational outreach visits. The top 10 most important strategies used, with their frequency and ease of use ratings are presented in Table 2.

**Table 2:** Heat map corresponding to importance, frequency, and ease of use for most important TNP strategies.

TNP Implementation Strategy	Strategy Definition	Importance Mean Definition	Frequency Mean Definition	Ease to Develop/Use Mean Definition
**Facilitation**	A process of interactive problem solving and support that occurs in a context of a recognized need for improvement and a supportive interpersonal relationship	**5** **Very Important**	**4.7** **1-3x/wk**	**2.7** **Less Easy**
**Centralize technical assistance**	Develop and use a centralized system to deliver technical assistance focused on implementation issues	**4.8** **Important**	**3.7** **1-2x/mos**	**2.7** **Less Easy**
**Provide clinical supervision.**	Provide clinicians with ongoing supervision focusing on the innovations. Provide training for clinical supervisors who will supervise clinicians who provide the innovation.	**4.8** **Important**	**3.7** **1-2x/mos**	**2.7** **Less Easy**
**Organize clinician implementation team meetings.**	Develop and support teams of clinicians who are implementing the innovation and give them protected time to reflect on the implementation effort, share lessons learned, and support one another’s learning.	**4.8** **Important**	**3.0** **1-2x/yr**	**2.7** **Less Easy**
**Use data experts.**	Involve, hire, and/or consult experts to inform management on the use of data generated by implementation efforts.	**4.7** **Important**	**3.0** **1-2x/mos**	**3.5** **Neutral**
**Assess for readiness and identify barriers and facilitators.**	Assess various aspects of an organization to determine its degree of readiness to implement, barriers that may impede implementation, and strengths that can be used in the implementation effort.	**4.7** **Important**	**2.7** **1-2x/yr**	**2.2** **Less Easy**
**Develop educational materials.**	Develop and format manuals, toolkits, and other supporting materials in ways that make it easier for stakeholders to learn about the innovation and for clinicians to learn how to deliver the clinical innovation.	**4.7** **Important**	**2.2** **1-2x/yr**	**2.5** **Less Easy**
**Conduct educational outreach visits**	Have a trained person meet with providers in their practice settings to educate providers about the clinical innovation, with the intent of changing the provider’s practice.	**4.7** **Important**	**2.0** **1-2x/yr**	**3.2** **Neutral**
**Fund and contract for the clinical innovation.**	Governments and other payers of services issue requests for proposals to deliver the innovation, use contracting processes to motivate providers to deliver the clinical innovation, and develop new funding formulas that make it more likely that providers will deliver the innovation.	**4.7** **Important**	**2.0** **1-2x/yr**	**3.0** **Neutral**
**Develop and implement tools for quality monitoring.**	Develop a formal implementation blueprint that includes all goals and strategies.	**4.7** **Important**	**2.0** **1-2x/yr**	**2.2** **Less Easy**

In phase four, interviews with six members of the TNP implementation team, one physician champion, and one transitions nurse (n = 8) indicated the TNP clinical leads primarily provided facilitation, centralized technical assistance, and clinical supervision. The TNP data manager also provided centralized technical assistance. Barriers to successful use included limited engagement from physician champions, site staff turnover, lower technical ability of some transitions nurses and personality differences. Contributors to success were the full-time support from the TNP clinical leads, TNP training sessions, the clinical leads communication styles, and on-site clinical observations that occurred in year two of TNP at all sites. The targets of all three strategies were transitions nurses and physician champions. All three strategies were frequently utilized in the beginning of TNP, but use tapered over time.

Phase four results indicated the primary differences between the facilitation, centralized technical assistance, and provide clinical supervision strategies were the activities required to support each strategy. Key facilitation activities included TNP providing “hands on” support to build relationships to improve program effectiveness, logistical support, and the extensive TNP training program [23]. Key activities reported for centralized technical assistance included the creation of the TNP database, the ability for near-real time audit and feedback, hands-on technical support, and the creation of on-line shared folders and a TNP SharePoint site. Key activities reported for the clinical supervision strategy included the availability of the TNP clinical leads during on-site clinical observations and through email, video, and phone.

## Discussion

TNP is an evidence-based intervention that positively impacted the health and safety of rural Veterans [7]. The implementation strategy postmortem method was developed to evaluate TNP implementation strategy fidelity and adaptations during the sustainment phase of the program. The postmortem approach identified 45 implementation strategies deployed in TNP, including the four that were identified *a priori*. This finding demonstrates the complexity of implementing a national care coordination intervention in multiple sites and the interdependence of various implementation strategies [24].

The postmortem method is an iterative, mixed method and multi-stakeholder approach that increased our understanding of the work that supported implementation of TNP across 11 VA medical centers over three years [25]. The initial research question “Which strategies were used” was best answered through qualitative methods (e.g., text analysis). The next question, “When were they used” was best answered using a quantitative approach due to the interest in collecting multiple perspectives. The final question, “Who and what approaches supported implementation” required a qualitative approach (e.g., interviews) to allow for an in-depth examination and contextualization of the previous findings. This approach allowed for meaningful inferences of the qualitative-quantitative-qualitative data [8], though required time and analytic skills. Future users of the postmortem method will benefit from the survey and interview tools provided in this paper, making the retrospective assessment of implementation fidelity and adaptations less cumbersome and time consuming.

Benefits of the multi-phase postmortem approach included insights into which TNP strategies were used, when, and with what purpose. We learned that many more implementation strategies were used during the 3-year study then were outlined in the TNP grant and none of the strategies were rated as very easy or easy to use or develop. We learned the implementation strategies were strategically introduced and discontinued across the QUERI Roadmap stages and the TNP clinical leads and database manager played a critical role in the success of TNP. This helped explain TNPs impact on increasing access to care and reducing mortality for high-risk Veterans [7]. Further, the postmortem findings identified the most important implementation strategies from the perspective of the implementation team and clinical staff.

Interactive assistance, which is a multifaceted strategy provided through facilitation, centralized technical assistance, and clinical supervision, aims to build trust and provide support to clinical teams [18]. This study revealed the specific aspects of interactive assistance and the many players and activities that enabled successful implementation of TNP and the positive outcomes for Veterans [7]. The interactive assistance implementation approach was strengthened by the hiring of full-time clinical leads who received ongoing communication and relationship training, [26] and the hiring of a database manager to create, adapt and deliver technical assistance to sites. The clinical supervision strategy for TNP was delivered at onboarding and annual intervention training, and through the TNP nurse learning community [27]. The postmortem method provided more than evaluation of implementation strategy fidelity and adaptations. The retrospective evaluation provided key data on the importance of having well-trained staff with dedicated time to devote to implementation of the intervention.

The postmortem method revealed variation in the number of implementation strategies used at different phases of the project across TNP sites, with most strategies used in the pre-implementation phase followed by the implementation, and sustainment phases. The emphasis on pre-implementation strategies can be explained by the TNP team’s efforts to build strong relationships with clinical and operational partners, engage stakeholders, diagnose local capabilities, assess barriers to implementation, and identify meaningful measures to inform the evaluation plan at every site to ensure successful implementation [3]. Implementation and sustainment efforts were streamlined once TNP was adapted to local contextual needs. This finding is important as we reflect on the design of implementation studies. Implementation strategies, like interventions, should dynamically change as the study progresses through its natural phases and the context and related barriers, facilitators, and priorities change. Most implementation studies, including TNP, are designed with static implementation strategies which does not align with how research projects and implementation of programs happen in the real world [5, 27].

Similar findings to our study were reported in the context of implementation of a new treatment for Hepatitis C virus in a national sample of VA medical centers [28]. In this 2017 study, researchers found that VA medical centers used, on average, 25 implementation strategies to support adoption of a new Hepatitis C treatment. Interestingly, the number of implementation strategies used was correlated with the number of Veterans who started the treatment at each site. Comparable to our findings, they also reported the ERIC implementation strategies “provide interactive assistance” and “develop stakeholder interrelationships” had the most significant impact on medication treatment. Of interest, when implementation strategies were tracked over time for Hepatitis C treatment starts, the strategies associated with treatment starts during pre-implementation (i.e., “training/educating,” “interactive assistance,” and “building stakeholder interrelationships”) were different than the strategies associated with treatment starts during the implementation phase (i.e., “evaluative and iterative” and “adapting and tailoring”) [29]. These findings match the TNP experience in that multi-faceted implementation strategies, iteratively selected and tailored to program needs, can have meaningful positive outcomes for patients [30].

The postmortem findings should be interpreted with some limitations in mind. The postmortem approach required retrospective identification, ranking, and specification of implementation strategies by staff who had extended relationships with the study investigators. There is a risk the results were influenced by recall and desirability biases. To minimize these biases multiple data sources (e.g., member checking, surveys, interviews) were used [31]. Further, the small sample and inclusion of perspectives from some, but not all TNP clinical sites, limits our understanding of every TNP members perception of the implementation strategies.

## Conclusions

Evaluating implementation strategy fidelity and adaptations are important activities to support implementation, sustainment and scale up of evidence-based interventions. The postmortem method described in this paper fills a critical gap. Implementation studies should be required, as part of good scientific practice, to evaluate the implementation strategies they used post-hoc. The method is an accessible and pragmatic approach to gather insights into the most important and frequently used implementation strategies deployed to support complex interventions. Moving forward, implementation scientists and healthcare teams can use the postmortem method and tools to enhance the implementation of evidence-based interventions across various patient safety projects (e.g. healthcare-acquired conditions, pain management) related to improving the health, safety, and equity of healthcare.

## Supporting information

S1 File ERIC implementation strategies. (PDF)

S2 File TNP implementation strategy survey. (DOCX)

S3 File Interview guide: TNP implementation strategies. (DOCX)

## Abbreviations

CI: Confidence Interval
ERIC: Expert Recommendations for Implementing Change
FRAME-IS: Framework for Reporting Adaptations and Modifications to Evidence-based Implementation Strategies
LISTS: Longitudinal Implementation Strategies Tracking System
QUERI: Quality Enhancement Research Initiative
RE-AIM: Reach, Effectiveness, Adoption, Implementation, Maintenance Framework
TNP: rural Transitions Nurse Program
VA: Veterans Health Administration
