# Supplementary material for: An implementation strategy postmortem method developed in the VA rural Transitions Nurse Program to inform spread and scale-up
Source: PLoS One. 2024 Mar 8;19(3):e0298552. doi: 10.1371/journal.pone.0298552 (PMC10923440; doi:10.1371/journal.pone.0298552)
Supplement: S1 File — (PDF) [file pone.0298552.s001.pdf]

Supplemental Table 1: ERIC Implementation Strategies and Definitions	
**Individual Strategy Title**	**ERIC Definition**
**Access new funding.**	Access new or existing money to facilitate the implementation.
**Alter incentive/allowance structures.**	Work to incentivize the adoption and implementation of the clinical innovation.
**Alter patient/consumer fees.**	Create fee structures where patients/consumers pay less for preferred treatments (the clinical innovation) and more for less-preferred treatments.
**Assess for readiness and identify barriers and facilitators.**	Assess various aspects of an organization to determine its degree of readiness to implement, barriers that may impede implementation, and strengths that can be used in the implementation effort.
**Audit and provide feedback.**	Collect and summarize clinical performance data over a specified time period and give it to clinicians and administrators to monitor, evaluate, and modify provider behavior.
**Build a coalition.**	Recruit and cultivate relationships with partners in the implementation effort.
**Capture and share local knowledge.**	Capture local knowledge from implementation sites on how implementers and clinicians made something work in their setting and then share it with other sites.
**Centralize technical assistance**	Develop and use a centralized system to deliver technical assistance focused on implementation issues
**Change accreditation or membership requirements.**	Strive to alter accreditation standards so that they require or encourage use of the clinical innovation. Work to alter membership organization requirements so that those who want to affiliate with the organization who are encouraged or required to use the clinical innovation.
**Change liability laws.**	Participate in liability reform efforts that make clinicians more willing to deliver the clinical innovation.
**Change physical structure and equipment.**	Evaluate current configurations and adapt, as needed, the physical structure and/or equipment (e.g., changing the layout of a room, adding equipment) to best accommodate the targeted innovation.
**Change record systems.**	Change record systems to allow better assessment of implementation or clinical outcomes.
**Change service sites.**	Change the location of clinical service sites to increase access.
**Conduct cyclical small tests of change.**	Implement changes in a cyclical fashion using small tests of change before taking changes systemwide. Tests of change benefit from systematic measurement, and results of the tests of change are studied for insights on how to do better. This process continues serially over time, and refinement is added with each cycle.

**Conduct educational meetings.**	Hold meetings targeted toward different stakeholder groups (e.g., providers, administrators, other organizational stakeholders, and community, patient/consumer, and family stakeholders) to teach them about the clinical innovation.
**Conduct educational outreach visits**	Have a trained person meet with providers in their practice settings to education providers about the clinical innovation with the intent of changing the providers practice.
**Conduct local consensus discussions.**	Include local providers and other stakeholders in discussions that address whether the chosen problem is important and whether the clinical innovation to address it is appropriate.
**Conduct local needs assessment**	Collect and analyze data related to the need for innovation
**Conduct ongoing training.**	Plan for and conduct training in the clinical innovation in an ongoing way.
**Create a learning collaborative.**	Facilitate the formation of groups of providers or provider organizations and foster a collaborative learning environment to improve implementation of the clinical innovation.
**Create new clinical teams.**	Change who serves on the clinical team, adding different disciplines and different skills to make it more likely that the clinical innovation is delivered (or is more successfully delivered).
**Create or change credentialing and/or licensure standards.**	Create an organization that certifies clinicians in the innovation or encourage an existing organization to do so. Change governmental professional certification or licensure requirements to include delivering the innovation. Work to alter continuing education requirements to shape professional practice toward the innovation.
**Develop a formal implementation blueprint.**	Develop a formal implementation blueprint that includes all goals and strategies. The blueprint should include the following: 1) aim/purpose of the implementation; 2) scope of the change (e.g., what organizational units are affected); 3) timeframe and milestones; and 4) appropriate performance/progress measures. Use and update this plan to guide the implementation effort over time.
**Develop academic partnerships.**	Partner with a university or academic unit for the purposes of shared training and bringing research skills to an implementation project.
**Develop an implementation glossary.**	Develop and distribute a list of terms describing the innovation, implementation, and stakeholders in the organizational change.
**Develop and implement tools for quality monitoring.**	Develop, test, and introduce into quality-monitoring systems the right input-the appropriate language, protocols, algorithms, standards, and measures (of processes, patient/consumer outcomes, and implementation outcomes) that are often specific to the innovation being implemented.

**Develop and organize quality monitoring systems.**	Develop and organize systems and procedures that monitor clinical processes and/or outcomes for the purpose of quality assurance and improvement.
**Develop disincentives.**	Provide financial disincentives for failure to implement or use the clinical innovations.
**Develop educational materials.**	Develop and format manuals, toolkits, and other supporting materials in ways that make it easier for stakeholders to learn about the innovation and for clinicians to learn how to deliver the clinical innovation.
**Develop resource sharing agreements.**	Develop partnerships with organizations that have resources needed to implement the innovation.
**Distribute educational materials.**	Distribute educational materials (including guidelines, manuals, and toolkits) in person, by mail, and/or electronically.
**Facilitate relay of clinical data to providers.**	Provide as close to real-time data as possible about key measures of process/outcomes using integrated modes/channels of communication in a way that promotes use of the targeted innovation.
**Facilitation**	A process of interactive problem solving and support that occurs in a context of a recognized need for improvement and a supportive interpersonal relationship
**Fund and contract for the clinical innovation.**	Governments and other payers of services issue requests for proposals to deliver the innovation, use contracting processes to motivate providers to deliver the clinical innovation, and develop new funding formulas that make it more likely that providers will deliver the innovation.
**Identify and prepare champions**	Identify and prepare individuals who dedicate themselves to supporting, marketing, and driving through an implementation, overcoming indifference or resistance to that the intervention may provoke in an organization.
**Identify early adopters.**	Identify early adopters at the local site to learn from their experiences with the practice innovation.
**Increase demand.**	Attempt to influence the market for the clinical innovation to increase competition intensity and to increase the maturity of the market for the clinical innovation.
**Inform local opinion leaders.**	Inform providers identified by colleagues as opinion leaders or "educationally influential" about the clinical innovation in the hopes that they will influence colleagues to adopt it.
**Intervene with patients/consumers to enhance uptake and adherence.**	Develop strategies with patients to encourage and problem solve around adherence.

**Involve executive boards.**	Involve existing governing structures (e.g., boards of directors, medical staff boards of governance) in the implementation effort, including the review of data on implementation processes.
**Involve patients/consumers and family members.**	Engage or include patients/consumers and families in the implementation effort.
**Make billing easier.**	Make it easier to bill for the clinical innovation.
**Make training dynamic.**	Vary the information delivery methods to cater to different learning styles and work contexts, and shape the training in the innovation to be interactive.
**Mandate change.**	Have leadership declare the priority of the innovation and their determination to have it implemented.
**Model and simulate change.**	Model or simulate the change that will be implemented prior to implementation.
**Obtain and use patients/consumers and family feedback.**	Develop strategies to increase patient/consumer and family feedback on the implementation effort.
**Obtain formal commitments**	Obtain written commitments from key partners that state what they will do to implement the innovation.
**Organize clinician implementation team meetings.**	Develop and support teams of clinicians who are implementing the innovation and give them protected time to reflect on the implementation effort, share lessons learned, and support one another's learning.
**Place innovation on fee for service lists/formularies.**	Work to place the clinical innovation on lists of actions for which providers can be reimbursed (e.g., a drug is placed on a formulary, a procedure is now reimbursable).
**Prepare patients/consumers to be active participants.**	Prepare patients/consumers to be active in their care, to ask questions, and specifically to inquire about care guidelines, the evidence behind clinical decisions, or about available evidence-supported treatments.
**Promote adaptability.**	Identify the ways a clinical innovation can be tailored to meet local needs and clarify which elements of the innovation must be maintained to preserve fidelity.
**Promote network weaving.**	Identify and build on existing high-quality working relationships and networks within and outside the organization, organizational units, teams, etc. to promote information sharing, collaborative problem solving, and a shared vision/goal related to implementing the innovation.
**Provide clinical supervision.**	Provide clinicians with ongoing supervision focusing on the innovations. Provide training for clinical supervisors who will supervise clinicians who provide the innovation.

**Provide local technical assistance.**	Develop and use a system to deliver technical assistance focused on implementation issues using local personnel.
**Provide ongoing consultation.**	Provide ongoing consultation with one or more experts in the strategies used to support implementing the innovation.
**Purposefully reexamine the implementation**	Monitor progress and adjust clinical practices and implementation strategies to continuously improve the quality of care
**Recruit, designate, and train for leadership.**	Recruit, designate, and train leaders for the change effort.
**Remind clinicians.**	Develop reminder system designed to help clinicians to recall information and/or prompt them to use the clinical innovation.
**Revise professional roles.**	Shift and revise roles among professionals who provide care, and redesign job characteristics.
**Shadow other experts.**	Provide ways for key individuals to directly observe experienced people engage with or use the targeted practice change/innovation.
**Stage implementation scale up**	Phase implementation efforts by starting with small pilots or demonstration projects and gradually move to a systemwide roll-out.
**Start a dissemination organization.**	Identify or start a separate organization that is responsible for disseminating the clinical innovation. It could be a for-profit or non-profit organization.
**Tailor strategies.**	Tailor the implementation strategies to address barriers and leverage facilitators that were identified through earlier data collection.
**Use advisory boards and workgroups.**	Create and engage a formal group of multiple kinds of stakeholders to provide input and advice on implementation efforts and to elicit recommendations for improvements.
**Use an implementation advisor.**	Seek guidance from experts in implementation.
**Use capitated payments.**	Pay providers or care systems a set amount per patient/consumer for delivering clinical care.
**Use data experts.**	Involve, hire, and/or consult experts to inform management on the use of data generated by implementation efforts.
**Use data warehousing techniques.**	Integrate clinical records across facilities and organizations to facilitate implementation across systems.
**Use mass media.**	Use media to reach large numbers of people to spread the word about the clinical innovation.
**Use other payment schemes.**	Introduce payment approaches (in a catch-all category).
**Use train-the-trainer strategies.**	Train designated clinicians or organizations to train others in the clinical innovation.

**Visit other sites.**	Visit sites where a similar implementation effort has been considered successful.
**Work with educational institutions.**	Encourage educational institutions to train clinicians in the innovation.
