# Supplementary material for: An implementation strategy postmortem method developed in the VA rural Transitions Nurse Program to inform spread and scale-up
Source: PLoS One. 2024 Mar 8;19(3):e0298552. doi: 10.1371/journal.pone.0298552 (PMC10923440; doi:10.1371/journal.pone.0298552)

**TNP Implementation Strategy Survey**

You are being asked to participate in this survey because you played an important role in the implementation of the rural Transitions Nurse Program (TNP).

In the original TNP grant, we identified a handful of implementation strategies that would be used to support implementation. While writing the annual reports for Office of Rural Health, the group felt we used more implementation strategies than we had described in the TNP grant.

To understand the breadth of strategies used in TNP, we reviewed the 73 discrete implementation strategies outlined by Powell et al (A refined compilation of implementation strategies: results from the Expert Recommendations for Implementing Change project. *Implementation Science, 2015: 10*(1), 21) The TNP team identified a total of 44 individual implementation strategies that were used in TNP.

This survey lists the 45 implementation strategies and includes an example of how it was used in TNP. For each strategy, please rate its importance, frequency with which it was used, and the ease of use (i.e., feasibility). We are asking for your opinion, there are no wrong answers.

Should you have any comments or questions, please feel free to contact us.

Thank you for your time and contribution to this process.

Heather Gilmartin Mary Nunnery Borsika Rabin

[Heather.gilmartin@va.gov](mailto:Heather.gilmartin@va.gov) [mary.nunnery@va.gov](mailto:mary.nunnery@va.gov) [barabin@health.ucsd.edu](mailto:barabin@health.ucsd.edu)

Section 1: Importance

1. Name:
2. What is/was your role on TNP?

**How important** were the following implementation strategies in the implementation of TNP?

| **How important was….** | **TNP Definition** | **Not important** | **Less Important** | **Neutral** | **Important** | **Very important** |
| --- | --- | --- | --- | --- | --- | --- |
| Centralized technical assistance | In TNP, we developed and used the Denver-based team as a core group to deliver technical assistance focused on implementation issues |  |  |  |  |  |
| Facilitation | In TNP, Lynette, Ashlea and the evaluation team created supportive interpersonal relationships with sites and provided interactive problem solving for sites that requested assistance or when there was a recognized need for improvement |  |  |  |  |  |
| Provide clinical supervision | In TNP, Lynette provided the transitions nurses with ongoing supervision focusing on the TNP intervention. Lynette and Bob provided training for the site champions who supervised the TNs regarding the TNP intervention. |  |  |  |  |  |
| Provide local technical assistance. | In TNP, we developed and used site champions and IT staff as local technical help during implementation. |  |  |  |  |  |
| Assess for readiness and identify barriers and facilitators. | In TNP, pre-implementation assessment of various aspects of an organization was conducted to determine a sites degree of readiness to implement, barriers that may impede implementation, and strengths that can be used in the implementation effort. |  |  |  |  |  |
| Audit and provide feedback. | In TNP, we collected and summarized clinical performance data using a data dashboard. We reported results to sites through the data dashboard and during monthly meetings to monitor, evaluate, and modify provider behavior. |  |  |  |  |  |
| **How important was….** | **TNP Definition** | **Not important** | **Less Important** | **Neutral** | **Important** | **Very important** |
| Conduct local needs assessment | In TNP, local organizational data were collected and analyzed to identify the need for TNP at each site. |  |  |  |  |  |
| Develop and organize quality monitoring systems. | In TNP, we developed and organized a reporting system (i.e. data dashboard) and created policies and procedures to monitor TNP processes, Veteran enrollment and satisfaction for the purpose of quality assurance and improvement. |  |  |  |  |  |
| Purposefully reexamine the implementation | In TNP, we monitored progress and adjusted clinical practices (i.e. eligibility criteria) and implementation strategies (i.e. frequency of learning collaborative meetings) to continuously improve the quality of care |  |  |  |  |  |
| Obtain and use patients/consumers and family feedback. | In TNP, we developed strategies (i.e. Veteran feedback form and IVR system to capture Veteran satisfaction) to increase patient/consumer and family feedback on the implementation effort. |  |  |  |  |  |
| Develop and implement tools for quality monitoring. | In TNP, we developed a formal implementation blueprint (e.g. toolkits) that included all goals and strategies. The toolkits were used to guide the implementation efforts for both cohorts. |  |  |  |  |  |
| Stage implementation scale up | In TNP, we phased implementation by starting with a pilot in Denver, then at 6 sites in year 1 and 5 additional sites in year 2. |  |  |  |  |  |
| Promote adaptability. | In TNP, we encouraged sites to tailor eligibility criteria and adapt implementation. Fidelity to the four step TNP intervention was a requirement. To capture adaptations, we created a section of the data dashboard for sites to enter this information. We also conducted adaptation interviews. |  |  |  |  |  |

| **How important was….** | **TNP Definition** | **Not important** | **Less Important** | **Neutral** | **Important** | **Very important** |
| --- | --- | --- | --- | --- | --- | --- |
| Tailor strategies. | In TNP, we tailored the implementation strategies (i.e. training timing, content, program materials) to address barriers and leverage facilitators that were identified through pre-implementation site visits. |  |  |  |  |  |
| Use data experts. | In TNP, we involved, hired and consulted with data experts (i.e. Meg, Anne, Ted, Dave) to inform management on the use of data generated by implementation efforts. |  |  |  |  |  |
| Use data warehousing techniques. | In TNP, we integrated clinical records across facilities and organizations through the use of a TNP data dashboard to facilitate implementation across systems. |  |  |  |  |  |
| Conduct educational meetings. | In TNP, we conducted educational meeting targeted to nurses, site champions, and site leadership to teach them TNP. |  |  |  |  |  |
| Conduct ongoing training. | In TNP, we conducted initial and annual training to support TNP implementation and sustainment. |  |  |  |  |  |
| Create a learning collaborative. | In TNP, we created a learning collaborative by fostering relationships and hosting regular meetings with transitions nurses and champions to improve implementation of TNP |  |  |  |  |  |
| Develop educational materials. | In TNP, we developed toolkits, and other supporting materials so stakeholders could learn how to deliver TN and share information about TNP to colleagues, leadership and Veterans. |  |  |  |  |  |
| Distribute educational materials. | In TNP, educational materials (including guidelines, manuals, pamphlets, posters, PowerPoints and toolkits) were distributed in person, during on-site meetings and electronically. |  |  |  |  |  |

| **How important was….** | **TNP Definition** | **Not important** | **Less Important** | **Neutral** | **Important** | **Very important** |
| --- | --- | --- | --- | --- | --- | --- |
| Provide ongoing consultation. | In TNP, we provided ongoing consultation with qualitative and quantitative experts support implementing TNP. |  |  |  |  |  |
| Conduct educational outreach visits | In TNP, educational outreach visits were conducted (by Lynette, Ashlea, and site nurses) to educate providers about TNP, with the intent of changing the provider's practice. |  |  |  |  |  |
| Make training dynamic. | In TNP, we varied training methods to cater to different learning styles (i.e. lecture, fishbowl exercise, role play with standardized actors) and work contexts (i.e. in person, on-line), and shaped the trainings to be interactive. |  |  |  |  |  |
| Shadow other experts. | In TNP, we provided funds for transitions nurses to travel to other sites to directly observe experienced transitions nurses in practice. |  |  |  |  |  |
| Develop resource sharing agreements. | In TNP, we developed partnerships with Office of Rural Health and Office of Nursing Services that have resources needed to support implementation of TNP. |  |  |  |  |  |
| Facilitate relay of clinical data to providers. | In TNP, we provided as close to real-time data as possible about TNP processes, Veteran enrollment and satisfaction using the data dashboard and meetings and recently weekly emails. . |  |  |  |  |  |
| Revise professional roles. | In TNP we revised a traditional nurse care coordination role to one specific for high-risk rural Veterans transferring from hospital back to their rural PCP. |  |  |  |  |  |
| Remind clinicians. | In TNP, a data dashboard was developed to help clinicians recall information and prompt them to adhere to the TNP intervention and seek referrals from providers |  |  |  |  |  |
| **How important was….** | **TNP Definition** | **Not important** | **Less Important** | **Neutral** | **Important** | **Very important** |
| Conduct local consensus discussions | In TNP, local providers and other stakeholders were included in pre-implementation discussions to learn if TNP was appropriate for their setting and population. |  |  |  |  |  |
| Model and simulate change | In TNP we modeled the change in practice at the Denver site, then used that experience to support and simulate change in new sites. |  |  |  |  |  |
| Build a coalition | In TNP, we cultivated relationships with sites and partners in the implementation effort by hosting annual meetings in Denver that included social events. |  |  |  |  |  |
| Capture and share local knowledge. | In TNP, we captured local knowledge from implementation sites on how implementers and clinicians made something work in their setting during pre-implementation site visits and on learning collaborative calls. We then shared this information with all sites. |  |  |  |  |  |
| Develop academic partnerships. | In TNP, we partnered with the University of Colorado Center for Advancing Professional Excellence to create an evidence-based education program to support TNP implementation. |  |  |  |  |  |
| Identify and prepare champions | In TNP, we identified and prepared site champions who dedicated .125 FTE to support, market and drive implementation at their sites. |  |  |  |  |  |
| Inform local opinion leaders | In TNP, we supported site champions to identify opinion leaders for TNP in the hopes they will influence colleagues to refer Veterans and support sustainment. |  |  |  |  |  |

| **How important was….** | **TNP Definition** | **Not important** | **Less Important** | **Neutral** | **Important** | **Very important** |
| --- | --- | --- | --- | --- | --- | --- |
| Organize clinician implementation team meetings | In TNP, we developed and supported TNP teams implementing the program at their sites by giving them 1.0FTE. This provided them protected time to learn and train for the intervention, guide and reflect on the implementation effort, share lessons learned, attend implementation meetings and trainings, enter program data into the TNP database and support other transitions nurses. |  |  |  |  |  |
| Promote network weaving. | In TNP, we identified and built on working relationships and networks within the VA (i.e. presenting on national cyberseminars and phone calls) and outside the VA (i.e. attendance at AAACN), to promote information sharing, collaborative problem solving, and a shared vision/goal related to implementing TNP. |  |  |  |  |  |
| Recruit, designate, and train for leadership | In TNP, we provided training to transitions nurses and site champions on how to be leaders of change in local care coordination efforts. |  |  |  |  |  |
| Use an implementation advisor | In TNP, we received expert consultation from Russ Glasgow and Borsika Rabin – implementation experts. |  |  |  |  |  |
| Involve executive boards | In TNP, we involved local executive boards during pre-implementation to support implementation efforts. Site champions were encouraged to continue engagement with the C-suite during implementation. |  |  |  |  |  |

| **How important was….** | **TNP Definition** | **Not important** | **Less Important** | **Neutral** | **Important** | **Very important** |
| --- | --- | --- | --- | --- | --- | --- |
| Fund and contract for the clinical innovation | In TNP, we received grant funding from the Office of Rural Health to support implementation of TNP in 11 VA medical centers for three years. We did not engage contracting or develop new funding formulas to make it more likely that providers will deliver TNP. |  |  |  |  |  |
| Change record systems | In TNP, we changed the VA electronic medical record system by creating a TNP transitions of care standardized note that was sent to primary care clinics and hospitalists upon Veteran discharge. The purpose was to standardize communication of Veteran care processes and post-discharge needs to support Veteran health and safety. |  |  |  |  |  |
| Intervene with patients/consumers to enhance uptake and adherence | In TNP, we interviewed rural Veterans prior to and during implementation to develop strategies that centered on their needs and to promote adherence with post-discharge follow-up. |  |  |  |  |  |
| Prepare patients/consumers to be active participants | In TNP, we developed standardized patient information materials to encourage them to be active in their care, to ask questions, and specifically to inquire about TNP, post-discharge expectations, and available resources. |  |  |  |  |  |

Section 2: Frequency

**How frequently** were the following strategies used to implement TNP? (Please consider as an average for all TNP sites)

| **How frequently were….** | **TNP Definition** | **Never** | **1-2 times/ year** | **1-2 times/ month** | **1-3 times/ week** | **3+ times/ week** |
| --- | --- | --- | --- | --- | --- | --- |
| Centralized technical assistance | In TNP, we developed and used the Denver-based team as a core group to deliver technical assistance focused on implementation issues |  |  |  |  |  |
| Facilitation | In TNP, Lynette, Ashlea and the evaluation team created supportive interpersonal relationships with sites and provided interactive problem solving for sites that requested assistance or when there was a recognized need for improvement |  |  |  |  |  |
| Provide clinical supervision | In TNP, Lynette provided the transitions nurses with ongoing supervision focusing on the TNP intervention. Lynette and Bob provided training for the site champions who supervised the TNs regarding the TNP intervention. |  |  |  |  |  |
| Provide local technical assistance. | In TNP, we developed and used site champions and IT staff as local technical help during implementation. |  |  |  |  |  |
| Assess for readiness and identify barriers and facilitators. | In TNP, pre-implementation assessment of various aspects of an organization was conducted to determine a sites degree of readiness to implement, barriers that may impede implementation, and strengths that can be used in the implementation effort. |  |  |  |  |  |
| Audit and provide feedback. | In TNP, we collected and summarized clinical performance data using a data dashboard. We reported results to sites through the data dashboard and during monthly meetings to monitor, evaluate, and modify provider behavior. |  |  |  |  |  |

| **How frequently were….** | **TNP Definition** | **Never** | **1-2 times/ year** | **1-2 times/ month** | **1-3 times/ week** | **3+ times/ week** |
| --- | --- | --- | --- | --- | --- | --- |
| Conduct local needs assessment | In TNP, local organizational data were collected and analyzed to identify the need for TNP at each site. |  |  |  |  |  |
| Develop and organize quality monitoring systems. | In TNP, we developed and organized a reporting system (i.e. data dashboard) and created policies and procedures to monitor TNP processes, Veteran enrollment and satisfaction for the purpose of quality assurance and improvement. |  |  |  |  |  |
| Purposefully reexamine the implementation | In TNP, we monitored progress and adjusted clinical practices (i.e. eligibility criteria) and implementation strategies (i.e. frequency of learning collaborative meetings) to continuously improve the quality of care |  |  |  |  |  |
| Obtain and use patients/consumers and family feedback. | In TNP, we developed strategies (i.e. Veteran feedback form and IVR system to capture Veteran satisfaction) to increase patient/consumer and family feedback on the implementation effort. |  |  |  |  |  |
| Develop and implement tools for quality monitoring. | In TNP, we developed a formal implementation blueprint (e.g. toolkits) that included all goals and strategies. The toolkits were used to guide the implementation efforts for both cohorts. |  |  |  |  |  |
| Stage implementation scale up | In TNP, we phased implementation by starting with a pilot in Denver, then at 6 sites in year 1 and 5 additional sites in year 2. |  |  |  |  |  |
| Promote adaptability. | In TNP, we encouraged sites to tailor eligibility criteria and adapt implementation. Fidelity to the four step TNP intervention was a requirement. To capture adaptations, we created a section of the data dashboard for sites to enter this information. We also conducted adaptation interviews. |  |  |  |  |  |

| **How frequently were….** | **TNP Definition** | **Never** | **1-2 times/ year** | **1-2 times/ month** | **1-3 times/ week** | **3+ times/ week** |
| --- | --- | --- | --- | --- | --- | --- |
| Tailor strategies. | In TNP, we tailored the implementation strategies (i.e. training timing, content, program materials) to address barriers and leverage facilitators that were identified through pre-implementation site visits. |  |  |  |  |  |
| Use data experts. | In TNP, we involved, hired and consulted with data experts (i.e. Meg, Anne, Ted, Dave) to inform management on the use of data generated by implementation efforts. |  |  |  |  |  |
| Use data warehousing techniques. | In TNP, we integrated clinical records across facilities and organizations through the use of a TNP data dashboard to facilitate implementation across systems. |  |  |  |  |  |
| Conduct educational meetings. | In TNP, we conducted educational meeting targeted to nurses, site champions, and site leadership to teach them TNP. |  |  |  |  |  |
| Conduct ongoing training. | In TNP, we conducted initial and annual training to support TNP implementation and sustainment. |  |  |  |  |  |
| Create a learning collaborative. | In TNP, we created a learning collaborative by fostering relationships and hosting regular meetings with transitions nurses and champions to improve implementation of TNP |  |  |  |  |  |
| Develop educational materials. | In TNP, we developed toolkits, and other supporting materials so stakeholders could learn how to deliver TN and share information about TNP to colleagues, leadership and Veterans. |  |  |  |  |  |
| Distribute educational materials. | In TNP, educational materials (including guidelines, manuals, pamphlets, posters, PowerPoints and toolkits) were distributed in person, during on-site meetings and electronically. |  |  |  |  |  |

| **How frequently were….** | **TNP Definition** | **Never** | **1-2 times/ year** | **1-2 times/ month** | **1-3 times/ week** | **3+ times/ week** |
| --- | --- | --- | --- | --- | --- | --- |
| Provide ongoing consultation. | In TNP, we provided ongoing consultation with qualitative and quantitative experts support implementing TNP. |  |  |  |  |  |
| Conduct educational outreach visits | In TNP, educational outreach visits were conducted (by Lynette, Ashlea, and site nurses) to educate providers about TNP, with the intent of changing the provider's practice. |  |  |  |  |  |
| Make training dynamic. | In TNP, we varied training methods to cater to different learning styles (i.e. lecture, fishbowl exercise, role play with standardized actors) and work contexts (i.e. in person, on-line), and shaped the trainings to be interactive. |  |  |  |  |  |
| Shadow other experts. | In TNP, we provided funds for transitions nurses to travel to other sites to directly observe experienced transitions nurses in practice. |  |  |  |  |  |
| Develop resource sharing agreements. | In TNP, we developed partnerships with Office of Rural Health and Office of Nursing Services that have resources needed to support implementation of TNP. |  |  |  |  |  |
| Facilitate relay of clinical data to providers. | In TNP, we provided as close to real-time data as possible about TNP processes, Veteran enrollment and satisfaction using the data dashboard and meetings and recently weekly emails. . |  |  |  |  |  |
| Revise professional roles. | In TNP we revised a traditional nurse care coordination role to one specific for high-risk rural Veterans transferring from hospital back to their rural PCP. |  |  |  |  |  |
| Remind clinicians. | In TNP, a data dashboard was developed to help clinicians recall information and prompt them to adhere to the TNP intervention and seek referrals from providers |  |  |  |  |  |

| **How frequently were….** | **TNP Definition** | **Never** | **1-2 times/ year** | **1-2 times/ month** | **1-3 times/ week** | **3+ times/ week** |
| --- | --- | --- | --- | --- | --- | --- |
| Conduct local consensus discussions | In TNP, local providers and other stakeholders were included in pre-implementation discussions to learn if TNP was appropriate for their setting and population. |  |  |  |  |  |
| Model and simulate change | In TNP we modeled the change in practice at the Denver site, then used that experience to support and simulate change in new sites. |  |  |  |  |  |
| Build a coalition | In TNP, we cultivated relationships with sites and partners in the implementation effort by hosting annual meetings in Denver that included social events. |  |  |  |  |  |
| Capture and share local knowledge. | In TNP, we captured local knowledge from implementation sites on how implementers and clinicians made something work in their setting during pre-implementation site visits and on learning collaborative calls. We then shared this information with all sites. |  |  |  |  |  |
| Develop academic partnerships. | In TNP, we partnered with the University of Colorado Center for Advancing Professional Excellence to create an evidence-based education program to support TNP implementation. |  |  |  |  |  |
| Identify and prepare champions | In TNP, we identified and prepared site champions who dedicated .125 FTE to support, market and drive implementation at their sites. |  |  |  |  |  |
| Inform local opinion leaders | In TNP, we supported site champions to identify opinion leaders for TNP in the hopes they will influence colleagues to refer Veterans and support sustainment. |  |  |  |  |  |

| **How frequently were….** | **TNP Definition** | **Never** | **1-2 times/ year** | **1-2 times/ month** | **1-3 times/ week** | **3+ times/ week** |
| --- | --- | --- | --- | --- | --- | --- |
| Organize clinician implementation team meetings | In TNP, we developed and supported TNP teams implementing the program at their sites by giving them 1.0FTE. This provided them protected time to learn and train for the intervention, guide and reflect on the implementation effort, share lessons learned, attend implementation meetings and trainings, enter program data into the TNP database and support other transitions nurses. |  |  |  |  |  |
| Promote network weaving. | In TNP, we identified and built on working relationships and networks within the VA (i.e. presenting on national cyberseminars and phone calls) and outside the VA (i.e. attendance at AAACN), to promote information sharing, collaborative problem solving, and a shared vision/goal related to implementing TNP. |  |  |  |  |  |
| Recruit, designate, and train for leadership | In TNP, we provided training to transitions nurses and site champions on how to be leaders of change in local care coordination efforts. |  |  |  |  |  |
| Use an implementation advisor | In TNP, we received expert consultation from Russ Glasgow and Borsika Rabin – implementation experts. |  |  |  |  |  |
| Involve executive boards | In TNP, we involved local executive boards during pre-implementation to support implementation efforts. Site champions were encouraged to continue engagement with the C-suite during implementation. |  |  |  |  |  |

| **How frequently were….** | **TNP Definition** | **Never** | **1-2 times/ year** | **1-2 times/ month** | **1-3 times/ week** | **3+ times/ week** |
| --- | --- | --- | --- | --- | --- | --- |
| Fund and contract for the clinical innovation | In TNP, we received grant funding from the Office of Rural Health to support implementation of TNP in 11 VA medical centers for three years. We did not engage contracting or develop new funding formulas to make it more likely that providers will deliver TNP. |  |  |  |  |  |
| Change record systems | In TNP, we changed the VA electronic medical record system by creating a TNP transitions of care standardized note that was sent to primary care clinics and hospitalists upon Veteran discharge. The purpose was to standardize communication of Veteran care processes and post-discharge needs to support Veteran health and safety. |  |  |  |  |  |
| Intervene with patients/consumers to enhance uptake and adherence | In TNP, we interviewed rural Veterans prior to and during implementation to develop strategies that centered on their needs and to promote adherence with post-discharge follow-up. |  |  |  |  |  |
| Prepare patients/consumers to be active participants | In TNP, we developed standardized patient information materials to encourage them to be active in their care, to ask questions, and specifically to inquire about TNP, post-discharge expectations, and available resources. |  |  |  |  |  |

Section 3: Ease

**How easy were the following strategies to develop and use to support TNP implementation?**

**(Please consider the time, effort, people and materials required)**

| **How easy were….** | **TNP Definition** | **Not Easy** | **Less Easy** | **Neutral** | **Easy** | **Very Easy** |
| --- | --- | --- | --- | --- | --- | --- |
| Centralized technical assistance | In TNP, we developed and used the Denver-based team as a core group to deliver technical assistance focused on implementation issues |  |  |  |  |  |
| Facilitation | In TNP, Lynette, Ashlea and the evaluation team created supportive interpersonal relationships with sites and provided interactive problem solving for sites that requested assistance or when there was a recognized need for improvement |  |  |  |  |  |
| Provide clinical supervision | In TNP, Lynette provided the transitions nurses with ongoing supervision focusing on the TNP intervention. Lynette and Bob provided training for the site champions who supervised the TNs regarding the TNP intervention. |  |  |  |  |  |
| Provide local technical assistance. | In TNP, we developed and used site champions and IT staff as local technical help during implementation. |  |  |  |  |  |
| Assess for readiness and identify barriers and facilitators. | In TNP, pre-implementation assessment of various aspects of an organization was conducted to determine a sites degree of readiness to implement, barriers that may impede implementation, and strengths that can be used in the implementation effort. |  |  |  |  |  |
| Audit and provide feedback. | In TNP, we collected and summarized clinical performance data using a data dashboard. We reported results to sites through the data dashboard and during monthly meetings to monitor, evaluate, and modify provider behavior. |  |  |  |  |  |

| **How easy were….** | **TNP Definition** | **Not Easy** | **Less Easy** | **Neutral** | **Easy** | **Very Easy** |
| --- | --- | --- | --- | --- | --- | --- |
| Conduct local needs assessment | In TNP, local organizational data were collected and analyzed to identify the need for TNP at each site. |  |  |  |  |  |
| Develop and organize quality monitoring systems. | In TNP, we developed and organized a reporting system (i.e. data dashboard) and created policies and procedures to monitor TNP processes, Veteran enrollment and satisfaction for the purpose of quality assurance and improvement. |  |  |  |  |  |
| Purposefully reexamine the implementation | In TNP, we monitored progress and adjusted clinical practices (i.e. eligibility criteria) and implementation strategies (i.e. frequency of learning collaborative meetings) to continuously improve the quality of care |  |  |  |  |  |
| Obtain and use patients/consumers and family feedback. | In TNP, we developed strategies (i.e. Veteran feedback form and IVR system to capture Veteran satisfaction) to increase patient/consumer and family feedback on the implementation effort. |  |  |  |  |  |
| Develop and implement tools for quality monitoring. | In TNP, we developed a formal implementation blueprint (e.g. toolkits) that included all goals and strategies. The toolkits were used to guide the implementation efforts for both cohorts. |  |  |  |  |  |
| Stage implementation scale up | In TNP, we phased implementation by starting with a pilot in Denver, then at 6 sites in year 1 and 5 additional sites in year 2. |  |  |  |  |  |
| Promote adaptability. | In TNP, we encouraged sites to tailor eligibility criteria and adapt implementation. Fidelity to the four step TNP intervention was a requirement. To capture adaptations, we created a section of the data dashboard for sites to enter this information. We also conducted adaptation interviews. |  |  |  |  |  |

| **How easy were….** | **TNP Definition** | **Not Easy** | **Less Easy** | **Neutral** | **Easy** | **Very Easy** |
| --- | --- | --- | --- | --- | --- | --- |
| Tailor strategies. | In TNP, we tailored the implementation strategies (i.e. training timing, content, program materials) to address barriers and leverage facilitators that were identified through pre-implementation site visits. |  |  |  |  |  |
| Use data experts. | In TNP, we involved, hired and consulted with data experts (i.e. Meg, Anne, Ted, Dave) to inform management on the use of data generated by implementation efforts. |  |  |  |  |  |
| Use data warehousing techniques. | In TNP, we integrated clinical records across facilities and organizations through the use of a TNP data dashboard to facilitate implementation across systems. |  |  |  |  |  |
| Conduct educational meetings. | In TNP, we conducted educational meeting targeted to nurses, site champions, and site leadership to teach them TNP. |  |  |  |  |  |
| Conduct ongoing training. | In TNP, we conducted initial and annual training to support TNP implementation and sustainment. |  |  |  |  |  |
| Create a learning collaborative. | In TNP, we created a learning collaborative by fostering relationships and hosting regular meetings with transitions nurses and champions to improve implementation of TNP |  |  |  |  |  |
| Develop educational materials. | In TNP, we developed toolkits, and other supporting materials so stakeholders could learn how to deliver TN and share information about TNP to colleagues, leadership and Veterans. |  |  |  |  |  |
| Distribute educational materials. | In TNP, educational materials (including guidelines, manuals, pamphlets, posters, PowerPoints and toolkits) were distributed in person, during on-site meetings and electronically. |  |  |  |  |  |

| **How easy were….** | **TNP Definition** | **Not Easy** | **Less Easy** | **Neutral** | **Easy** | **Very Easy** |
| --- | --- | --- | --- | --- | --- | --- |
| Provide ongoing consultation. | In TNP, we provided ongoing consultation with qualitative and quantitative experts support implementing TNP. |  |  |  |  |  |
| Conduct educational outreach visits | In TNP, educational outreach visits were conducted (by Lynette, Ashlea, and site nurses) to educate providers about TNP, with the intent of changing the provider's practice. |  |  |  |  |  |
| Make training dynamic. | In TNP, we varied training methods to cater to different learning styles (i.e. lecture, fishbowl exercise, role play with standardized actors) and work contexts (i.e. in person, on-line), and shaped the trainings to be interactive. |  |  |  |  |  |
| Shadow other experts. | In TNP, we provided funds for transitions nurses to travel to other sites to directly observe experienced transitions nurses in practice. |  |  |  |  |  |
| Develop resource sharing agreements. | In TNP, we developed partnerships with Office of Rural Health and Office of Nursing Services that have resources needed to support implementation of TNP. |  |  |  |  |  |
| Facilitate relay of clinical data to providers. | In TNP, we provided as close to real-time data as possible about TNP processes, Veteran enrollment and satisfaction using the data dashboard and meetings and recently weekly emails. . |  |  |  |  |  |
| Revise professional roles. | In TNP we revised a traditional nurse care coordination role to one specific for high-risk rural Veterans transferring from hospital back to their rural PCP. |  |  |  |  |  |
| Remind clinicians. | In TNP, a data dashboard was developed to help clinicians recall information and prompt them to adhere to the TNP intervention and seek referrals from providers |  |  |  |  |  |

| **How easy were….** | **TNP Definition** | **Not Easy** | **Less Easy** | **Neutral** | **Easy** | **Very Easy** |
| --- | --- | --- | --- | --- | --- | --- |
| Conduct local consensus discussions | In TNP, local providers and other stakeholders were included in pre-implementation discussions to learn if TNP was appropriate for their setting and population. |  |  |  |  |  |
| Model and simulate change | In TNP we modeled the change in practice at the Denver site, then used that experience to support and simulate change in new sites. |  |  |  |  |  |
| Build a coalition | In TNP, we cultivated relationships with sites and partners in the implementation effort by hosting annual meetings in Denver that included social events. |  |  |  |  |  |
| Capture and share local knowledge. | In TNP, we captured local knowledge from implementation sites on how implementers and clinicians made something work in their setting during pre-implementation site visits and on learning collaborative calls. We then shared this information with all sites. |  |  |  |  |  |
| Develop academic partnerships. | In TNP, we partnered with the University of Colorado Center for Advancing Professional Excellence to create an evidence-based education program to support TNP implementation. |  |  |  |  |  |
| Identify and prepare champions | In TNP, we identified and prepared site champions who dedicated .125 FTE to support, market and drive implementation at their sites. |  |  |  |  |  |
| Inform local opinion leaders | In TNP, we supported site champions to identify opinion leaders for TNP in the hopes they will influence colleagues to refer Veterans and support sustainment. |  |  |  |  |  |

| **How easy were….** | **TNP Definition** | **Not Easy** | **Less Easy** | **Neutral** | **Easy** | **Very Easy** |
| --- | --- | --- | --- | --- | --- | --- |
| Organize clinician implementation team meetings | In TNP, we developed and supported TNP teams implementing the program at their sites by giving them 1.0FTE. This provided them protected time to learn and train for the intervention, guide and reflect on the implementation effort, share lessons learned, attend implementation meetings and trainings, enter program data into the TNP database and support other transitions nurses. |  |  |  |  |  |
| Promote network weaving. | In TNP, we identified and built on working relationships and networks within the VA (i.e. presenting on national cyberseminars and phone calls) and outside the VA (i.e. attendance at AAACN), to promote information sharing, collaborative problem solving, and a shared vision/goal related to implementing TNP. |  |  |  |  |  |
| Recruit, designate, and train for leadership | In TNP, we provided training to transitions nurses and site champions on how to be leaders of change in local care coordination efforts. |  |  |  |  |  |
| Use an implementation advisor | In TNP, we received expert consultation from Russ Glasgow and Borsika Rabin – implementation experts. |  |  |  |  |  |
| Involve executive boards | In TNP, we involved local executive boards during pre-implementation to support implementation efforts. Site champions were encouraged to continue engagement with the C-suite during implementation. |  |  |  |  |  |

| **How easy were….** | **TNP Definition** | **Not Easy** | **Less Easy** | **Neutral** | **Easy** | **Very Easy** |
| --- | --- | --- | --- | --- | --- | --- |
| Fund and contract for the clinical innovation | In TNP, we received grant funding from the Office of Rural Health to support implementation of TNP in 11 VA medical centers for three years. We did not engage contracting or develop new funding formulas to make it more likely that providers will deliver TNP. |  |  |  |  |  |
| Change record systems | In TNP, we changed the VA electronic medical record system by creating a TNP transitions of care standardized note that was sent to primary care clinics and hospitalists upon Veteran discharge. The purpose was to standardize communication of Veteran care processes and post-discharge needs to support Veteran health and safety. |  |  |  |  |  |
| Intervene with patients/consumers to enhance uptake and adherence | In TNP, we interviewed rural Veterans prior to and during implementation to develop strategies that centered on their needs and to promote adherence with post-discharge follow-up. |  |  |  |  |  |
| Prepare patients/consumers to be active participants | In TNP, we developed standardized patient information materials to encourage them to be active in their care, to ask questions, and specifically to inquire about TNP, post-discharge expectations, and available resources. |  |  |  |  |  |

THIS IS THE END OF THE SURVEY

THANK YOU


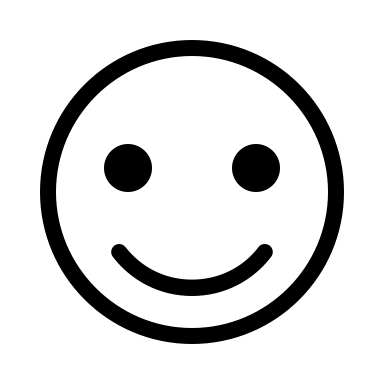

Supplement: S2 File — (DOCX) [file pone.0298552.s002.docx]
