# Supplementary material for: An implementation strategy postmortem method developed in the VA rural Transitions Nurse Program to inform spread and scale-up
Source: PLoS One. 2024 Mar 8;19(3):e0298552. doi: 10.1371/journal.pone.0298552 (PMC10923440; doi:10.1371/journal.pone.0298552)
Supplement: S3 File — (DOCX) [file pone.0298552.s003.docx]

**Interview Guide: TNP Implementation Strategies**

*For the Interviewer: Semi-structured interviews are open-ended to allow participants to freely express their answers to questions. Additional questions may be asked by the interviewers to follow-up or to clarify participants’ responses*

Hello, my name is __________ and I’m part of the TNP evaluation team. Thanks for speaking with me today. I am talking with you because of your knowledge about the implementation of the rural Transitions Nurse Program. I hope to learn about the implementation strategies used to support the program and any feedback you have for improving the process. This interview should take about 30 minutes.

To make sure I capture all of the information you give us, I would like to record our interview. Audio-files are encrypted and stored on a restricted VA server. You will not be identified as a participant in project reports or manuscripts. Your participation is voluntary. You may elect to end the interview at any time and let me know if you’d rather not answer a question. Before we begin, do you have any questions?

[Start Recording]

This is Mary Nunnery and I'm here with participant number _____; Today is ______ / ______ / _____.

Are you aware that you're being audio-recorded? *_____Yes _____No*

1. What is your role in TNP? ____________________________________________________________________________________________________________________________________________________________
   1. What does this work entail?

Now I’m going to ask you some details about three implementation strategies that the Denver team ranked as most important to the success of TNP.

The following strategies were found to be the three most important to implementing TNP.

- Facilitation
- Centralize Technical Assistance
- Provide Clinical Supervision

**Facilitation Section**

**The Definition of Facilitation is**: (Read definition to interviewee)

A process of interactive problem solving that occurs in a context of a recognized need for improvement and a supportive interpersonal relationship.

**Example**: In TNP, Lynette, Ashlea and the evaluation team created supportive interpersonal relationships with sites and provided interactive problem solving for sites that requested assistance or when there was a recognized need for improvement.

**1. How would you describe the facilitation provided during TNP?**

What were the key activities of facilitation?

Can you walk me through one facilitation experience?

**2. Do you think facilitation was an effective strategy to support implementation of TNP? (Interviewer to read responses)**

- Very effective
- Effective
- Neutral
- Less effective
- Not effective

**3. What, in your mind, contributed to the success of facilitation?**

Were there barriers to the use of facilitation?

Were there things that supported the use of facilitation?

**4. Who delivered facilitation primarily? Probe: Was it more than one person?** (actor)

Name:

Name:

Name:

**5. Who were the targets of facilitation? (Let the participant answer, then use the categories below as probes)**

- TNs
- Champions
- Veterans
- Office of Rural Health
- TNP Denver team ORH

**6. When in the process of implementing TNP was facilitation used?** (time)

- Before starting to enroll patients
- In the first couple months of enrollment
- Once the program was up and running
- When the program was moving to its end
- Throughout the whole program

**7. How often was facilitation used? (**frequency) **(Let the participant answer, then use the categories below as probes)**

- Every day
- 1-2 times a week or more
- 1-2 times a month or more
- Couple times a year

**8.**  **What TNP outcomes did facilitation impact? (Choose all that apply)**

- Reach of the TNP intervention to Veterans
- Adoption of the TNP intervention at sites
- Sustained use of the TNP intervention of sites
- Positive impact on Veteran health and safety (effectiveness)

**9. How easy was delivery (or receipt) of facilitation to support TNP implementation (consider the time, effort, people, and materials required).**

- Very easy
- Easy
- Neutral
- Less easy
- Not easy

**Probe as to why they rated it that way.**

**Centralize Technical Assistance Section**

**Now I am going to ask you some questions about the Centralize Technical Assistance provided during TNP**

**The Definition of Centralized Technical Assistance is**: (Read definition to interviewee):

Develop and use a centralized system to deliver technical assistance focused on implementation

issues.

**Example:** In TNP, we developed and used the Denver-based team as a core group to deliver technical assistance focused on implementation issues

**1. How would you describe the** **centralized technical assistance provided during TNP?**

What were the key components of **centralized technical assistance**?

Can you walk me through one **centralized technical assistance** experience?

**2. Do you think centralized technical assistance was an effective strategy to support implementation of TNP? (Interviewer to read responses)**

- Very effective
- Effective
- Neutral
- Less effective
- Not effective

**3.What, in your mind, contributed to the success of centralized technical assistance?**

Were there barriers to the use of **centralized technical assistance**?

Were there things that supported the use of **centralized technical assistance**?

**4. Who delivered centralized technical assistance primarily? Probe: Was it more than one person?** (actor)

Name:

Name:

Name:

**5. Who were the targets of centralized technical assistance? (Let the participant answer, then use the categories below as probes)**

- TNs
- Champions
- Veterans
- Office of Rural Health
- TNP Denver team ORH

**6. When in the process of implementing TNP was centralized technical assistance used?** (time)

- Before starting to enroll patients
- in the first couple months of enrollment
- Once the program was up and running
- Though out the whole program?

**7. How often was centralized technical assistance used? (**frequency) **(Let the participant answer, then use the categories below as probes)**

- Every day
- 1-2 times a week or more
- 1-2 times a month or more
- Couple times a year

**8.**  **What TNP outcomes did centralized technical assistance impact?**

- Reach of the TNP intervention to Veterans
- Adoption of the TNP intervention at sites
- Sustained use of the TNP intervention of sites
- Positive impact on Veteran health and safety (effectiveness)

**9. How easy was delivery (or receipt) of centralized technical assistance to support TNP implementation? (consider the time, effort, people, and materials required)**

- Very easy
- Easy
- Neutral
- Less easy
- Not easy

**Probe as to why they rated it that way.**

**Provide Clinical Supervision Section**

**Now I am going to ask you some questions about the provision of clinical supervision**

**The Definition of Provide Clinical Supervision is**: (Read definition to interviewee):

Provide clinicians with ongoing supervision focusing on the innovation. Provide training for

clinical supervisors who will supervise clinicians who provide the innovation.

**Example**: In TNP, Lynette provided the transitions nurses with ongoing supervision focusing on the TNP intervention. Lynette and Bob provided training for the site champions who supervised the TNs regarding the TNP intervention.

**1. How would you describe the** **clinical supervision provided during TNP?**

What were the key components of **clinical supervision provided during TNP**?

Can you walk me through one **clinical supervision** experience?

**2. Do you think** **clinical supervision was an effective strategy to support implementation of TNP? (Interviewer to read responses)**

- Very effective
- Effective
- Neutral
- Less effective
- Not effective

**3.What, in your mind, contributed to the success of providing clinical supervision?**

Were there barriers to the provision of clinical supervision?

Were there things that supported the provision of clinical supervision?

**4. Who delivered clinical supervision primarily? Probe: Was it more than one person?** (actor)

Name:

Name:

Name:

**5. Who were the targets of clinical supervision? (Let the participant answer, then use the categories below as probes)**

- TNs
- Champions
- Veterans
- Office of Rural Health
- TNP Denver team ORH

**6. When in the process of implementing TNP clinical supervision provided?** (time)

- Before starting to enroll patients
- in the first couple months of enrollment
- Once the program was up and running
- Though out the whole program?

**7. How often was clinical supervision provided? (**frequency) **(Let the participant answer, then use the categories below as probes)**

- Every day
- 1-2 times a week or more
- 1-2 times a month or more
- Couple times a year

**8.**  **What TNP outcomes did clinical supervision impact?**

- Reach of the TNP intervention to Veterans
- Adoption of the TNP intervention at sites
- Sustained use of the TNP intervention of sites
- Positive impact on Veteran health and safety (effectiveness)

**9. How easy was delivery (or receipt) of clinical supervision to support TNP implementation? (consider the time, effort, people, and materials required)**

- Very easy
- Easy
- Neutral
- Less easy
- Not easy

**Probe as to why they rated it that way.**

**Closing Questions:**

**Is there anything else you’d like to share about the TNP implementation strategies? Were there other strategies that you think were very important for the success of TNP?**

**Do you have any questions for me?**

Thank you for taking the time to participate in this interview. We really appreciate you sharing your experiences with implementing the TNP in the VA system.
